# Do patients with long-term side effects of cancer treatment benefit from general practitioner support? A literature review

**DOI:** 10.5334/ijic.1987

**Published:** 2015-06-08

**Authors:** Irene Ngune, Moyez Jiwa, Alexandra McManus, Jeff Hughes

**Affiliations:** Curtin University, Faculty of Health Sciences, Bentley, Perth, WA, Australia; Curtin University, Medical Education, Bentley, Perth, WA, Australia; Curtin University, Faculty of Health Sciences, Bentley, Perth, WA, Australia; Curtin University, School of Pharmacy, Bentley, Perth, WA, Australia

**Keywords:** cancer, primary health care, follow-up care, supportive care, integrated care

## Abstract

**Background:**

Alongside specialist cancer clinics, general practitioners have an important role in cancer patients’ follow-up care, yet no literature summarises the nature, extent and impact of their involvement. This paper addresses this issue through a review of the literature.

**Methods:**

Studies were sourced from six academic databases - AustHealth (*n* = 202), CINAHL (*n* = 500), the Cochrane Library (reviews and trials; *n* = 200), Embase (*n* = 368), PHCRIS (*n* = 132) and PubMed/Medline (*n* = 410). Studies that focused on interventions designed for patients receiving follow-up care and reported cancer care provided by a general practitioner delivered alongside specialist care were reviewed.

**Results:**

A total of 19 papers were identified as relevant for this review (3 randomised control trials; 4 cross-sectional, 5 cohort and 3 qualitative studies, and 3 systematic reviews). The reviewed studies indicated that providing general practitioner-led supportive interventions for post-treatment care of cancer patients is feasible and acceptable to patients. General practitioner involvement resulted in improved physical and psychosocial well-being of patients and continuity of care, especially for patients with concomitant health conditions.

**Conclusion:**

Involving general practitioners in post-treatment cancer care is beneficial to patients. However, proactive initiatives that encourage and facilitate patients to consult their general practitioner about their needs or symptoms of recurrence should be considered.

## Introduction

With improved survival of cancer patients following treatment, attention needs to be paid on the ongoing physical and psychosocial needs of this population [1]. Long-term physical problems and psychological morbidity such as anxiety [2], depression [3] and fear of recurrence [4], and [5, 6] social factors such as financial problems [7] and activity limitation [6, 8] continue to affect patients for many years following treatment.

Cancer patients are now living longer and many have co-existing health conditions [9, 10]. For example, about 50% of people with colorectal cancer are now living beyond 10 years after treatment [11] and between 30% and 60% of colorectal cancer survivors aged 70 years or older have other concomitant health conditions and are more likely to die of other causes [12]. As the survival of cancer patients improves, general practitioners will find that they occupy a larger proportion of their practices. Many of the cancer patients will have other medical issues and supporting people treated for cancer is going to become a significant feature of their practice. Also, with the increasing number of cancer patients accessing specialist cancer clinics, strategies that supplement these services will be required to support patients with long-term treatment side effects.

In Australia, cancer patients attend a general practitioner for multiple reasons [10]. Patients visit general practitioners for other health conditions and preventive health services such as screening, health promotion advice and vaccination [13]. Given that general practitioner services are a cornerstone of the Australian health system, there is potential for general practitioners to support cancer patients along with ongoing specialist care. Moreover, there is evidence that cancer patients first present to a general practitioner with cancer-related side effects or symptoms of recurrence, even with ongoing management by their specialist [14].

There is empirical evidence that a general practitioner-led follow-up model for cancer patients would be a safe and acceptable alternative to specialist follow-up especially for patients in remission [14]; however, oncologists still need to play a fundamental role in the management of these patients [15, 16]. Studies have shown that cancer patients who are followed by both oncologists and general practitioners receive better preventive health and cancer care compared to those who are managed by either of the specialities independently [13]. Also, some patients still value access to specialist services, especially in the early stages after treatment [15, 16].

To date, most of the literature reviews that report on interventions provided by a general practitioner concurrently with the specialist only provide details regarding organisation of patient care and flow of information between the specialist and the general practitioner [17–20]. Although these reviews offer recommendations regarding communication between the general practitioner and the specialist [18, 19, 21], patients’ outcome data regarding the issues addressed during patient visits with the general practitioner are limited [17].

Given these findings and the potential benefits of support that patients may receive by having a general practitioner involved in their cancer care, a literature review was conducted to assess care of patients in the context of ongoing specialist care and with particular reference to general practitioner involvement. This review focused on all types of cancer studies that met the inclusion criteria assessing integrated approaches to care for multiple cancer types.

## Aims

The aims of this literature review were to:

- Critically appraise the selected studies
- Describe proactive management of patients with long-term needs following cancer treatment, including surveillance for recurrence
- Describe the effectiveness of general practitioner support in post-treatment cancer care

## Methods

### Search strategy

A search strategy was developed to electronically source studies published in English from six academic databases - AustHealth, CINAHL, the Cochrane Online Library (reviews and trials), Embase, PHCRIS and PubMed/Medline. These were searched in January 2014 employing the following strategies using Medical Subject Headings (MeSH):

- Family practice [mh] OR primary health care [mh] or general practice AND
- Parallel care as Topic [mh] OR shared care as Topic [mh] OR cancer follow-up* care [mh] AND
- Evaluation research [mh]
- Randomised control trial [mh]
- Feasibility projects [mh]
- Various combinations (1 AND 2; 1 AND 3 AND 4; 1 AND 5; 2 AND 3; 2 AND 4; 2 AND 5)

### Eligibility of studies, types of participants and outcomes assessed

For inclusion, studies had to describe delivery of interventions by a general practitioner and care had to be delivered alongside specialist care. Studies that included adult cancer patients regardless of the site or stage of the disease were also eligible for review. For inclusion in the review, patients should have completed treatment for cancer. Also, studies evaluating general practitioners and patients perspectives to cancer shared care were included.

Since terms such as ‘shared care’, ‘complementary care’ and ‘parallel care’ were poorly standardised within the taxonomy and nomenclature of the electronic databases, the search strategy was kept as wide as possible. All papers in which such terms were stated were included in the review. In addition, the cancer follow-up phase was poorly defined with regards to when this period began. Hence, all studies in which patients had completed the indicated treatment were included in the review.

### Identification of studies

A total of 1802 papers were identified from the six academic databases - AustHealth (*n* = 202), CINAHL (*n* = 500), the Cochrane Library (reviews and trials; *n* = 200), Embase (*n* = 368), PHCRIS (*n* = 132) and PubMed/Medline (*n* = 410). Potentially relevant titles and abstracts of 533 references were reviewed using the following inclusion criteria:

- Study represents a research article (rather than a letter or commentary)
- Research context is primary care, that is, settings in which health practitioners were primary health physicians, family practice doctors or general practitioners
- Primary focus is to describe intervention(s) or evaluate care provided by a general practitioner alongside hospital care for patients who have completed cancer treatment

Titles and abstracts of articles identified through database searching and hand searching of the reference lists (*n* = 1802) were evaluated for relevance by three researchers (IN, MJ and AM). Potentially relevant articles (*n* = 533) were further subjected to a more detailed evaluation and 391 articles were excluded because they did not meet the inclusion criteria. The first author (IN) and two other reviewers (MJ and AM) independently reviewed the remaining 142 studies, with studies that included other models of post-treatment cancer care being excluded (n =123). In total, 19 studies were eligible to be included in the review.

**Figure 1.**
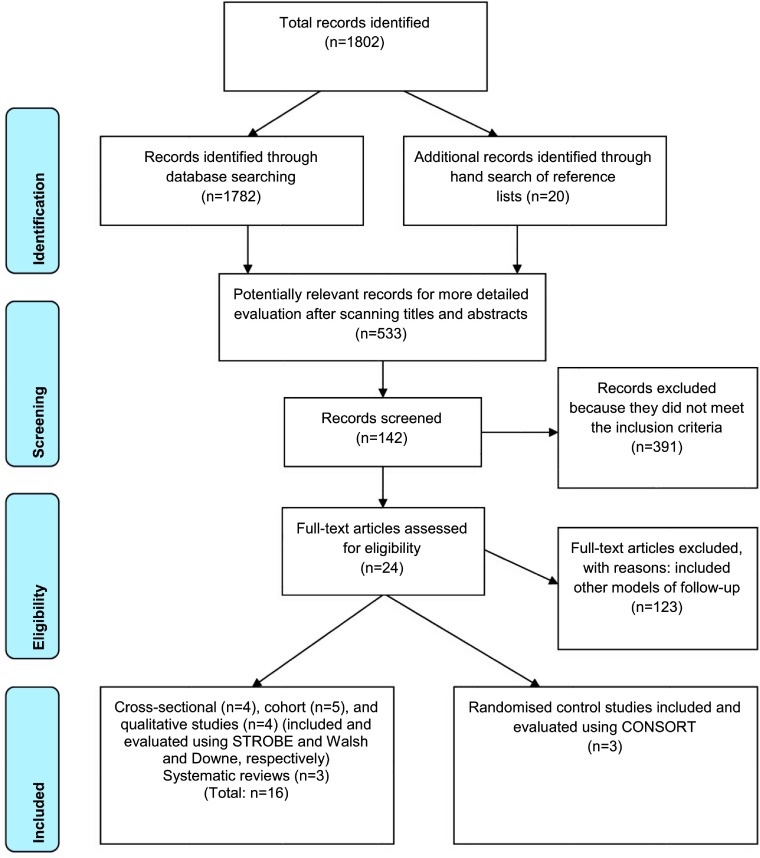
PRISMA flow diagram of the results of systematic review of general practitioner-led supportive care interventions

Schematic representation of the selection process of the articles included in this review is shown in Figure 1.

### Data extraction

One reviewer (IN) extracted articles and assessed the methodological quality of the studies using Consolidated Standards of Reporting Trials (CONSORT) [22] for randomised control trials, STrengthening the Reporting of OBservational studies in Epidemiology (STROBE) [23, 24] for cohort and cross-sectional studies and Walsh and Downe criteria [25] for qualitative studies.

CONSORT is an evidence-based, minimum set of recommendations for reporting randomised control trials. It offers a standard way for authors to prepare reports of trial findings, facilitating their complete and transparent reporting and aiding their critical appraisal and interpretation. The CONSORT statement comprises a 25-item checklist. The checklist items focus on reporting how the trial was designed, analysed and interpreted [22].

STROBE stands for an international, collaborative initiative of epidemiologists, methodologists, statisticians, researchers and journal editors involved in the conduct and dissemination of observational studies, with the common aim of strengthening the reporting of observational studies in epidemiology. STROBE stipulates a standard way for reporting study design, results and interpretation of cohort, case–control and cross-sectional studies. The checklist comprises of 22 items that offer a basis for evaluating observational studies [23, 24].

The Walsh and Downe recommendations are a set of iterative criteria that form a working framework for qualitative research appraisal. This checklist comprises of eight stages that aid critical appraisal of study designs, methodology, interpretation and transferability of results [25].

Selected articles were also reviewed by two other authors (MJ and AM) as a measure of inter-rater reliability. Differences in assessments by the reviewers were resolved by consensus when the full-text articles were reviewed.

The intervention, outcomes details and main conclusions were collected on a standard data sheet, which included type of study, author, data, sample size and participation rates (Table 1).

## Results

The reviewers reached a consensus on the remaining 19 articles, all of which were included in the review (Figure 1). There were three randomised control trials, five cohort studies, four cross-sectional studies, four qualitative studies and three systematic reviews. Due to variation in methodology of the studies and in how the findings were reported and analysed, a meta-analysis was not feasible even for studies with similar outcome measures. Additionally, all the studies included in this review were conducted in different countries with very different health care arrangements.

### Interventions and evaluation of general practitioner involvement

Studies where an intervention took place or was evaluated have been described below. In summary, 10 studies were based on a framework, which sought information from patients on care (psychological, physical and social care) provided by their general practitioner. Patients’ rehabilitation needs were assessed directly by the general practitioner or by cancer nurses/specialists and then relayed to the general practitioner in a letter.

In the Bergholdt et al. study, patients were invited for an interview about rehabilitation needs with a rehabilitation coordinator at the hospital. The information from the hospital was sent to the general practitioner about individual needs for rehabilitation and an encouragement of the general practitioner to contact the patient to offer his support with rehabilitation [26].

In the Holtedahl et al. study, cancer patients were invited to a 30-minute consultation with the patient's general practitioner, who was asked to let the patient tell about experiences as a cancer patient, and to tell the patient explicitly that she or he would be welcome to contact the general practitioner whenever there was a question or a problem related to their disease [27].

Two studies described a shared care programme between the general practitioner and the specialist: In the Nielsen et al. study, a discharge summary letter detailing patient's physical, psychological and social problems was posted to the general practitioner. The summary also contained information about what the oncologists expected the general practitioner to do, specific information about the patient's type of cancer, treatment plans and prognosis as well as general information about treatment of common side effects and pain. Names and phone numbers of the doctors and nurses responsible for the patients were also attached [28].

In the Hall et al. study, patients were asked to attend general practitioners for follow-up appointments. Follow-up protocols and a system of specialist support were sent to the general practitioners by the treating specialist. General practitioners were given an opportunity to shadow specialists as they conducted follow-up appointment at the hospital [29].

In the Sisler et al. study, colorectal cancer survivors who were sent a survey assessing their perceptions of continuity of care around the time of discharge from the cancer centre. Health-related quality of life was also assessed as many patients stated they had seen a general practitioner during their survivorship care [30].

Bowman et al. assessed primary care provider involvement in key activities measured by cancer survivors reports: if primary care providers discussed with patients cancer-related problems and if these discussions resulted in tests and procedures [31].

In five other studies, patients completed a survey on or data were analysed on either of the following: number of visits to the family physician in the prior year; family physician's, specialist's and oncology team's responsibility for cancer care; family physician's involvement in their care; perceived family physician's actual and expected roles in various aspects of care (coordination, psychosocial support, information transmission, symptom relief, preventive health); and the family physician pattern of care [32–36].

### Critical appraisal of the studies

#### Recruitment, randomisation and methods

Three randomised control trials with general practitioner interventions were identified [26–28]. All three studies fulfilled at least 22 of the 25 items in CONSORT and provided background details about the study objectives, eligible participants and outcomes of interest. Reporting of the randomisation process was generally poor in most of the studies and details of the allocation concealment were not fully provided. Strategies used to generate allocation sequences were only fully described by Bergholdt et al. [26] and Holtedahl et al. [27].

Of the five cohort and four cross-sectional studies, no studies fulfilled all criteria of the STROBE statement [23, 24]. Six studies [30, 32, 33, 35, 37, 38] provided clear information regarding the participants’ eligibility criteria, study setting, locations and relevant dates, including periods of recruitment. In all the nine studies, descriptions of the study methods were often sparse and/or were either missing or partially satisfied. Details of the studies’ appraisal using CONSORT and STROBE (randomised control trials and observational studies, respectively) have been summarised in Tables 2 and 3.

For the four qualitative studies [29, 31, 41, 42], nearly all criteria outlined by Walsh and Downe [25] were met. The studies were contextualised by existing literature, details of the methods/design were consistent with research intent, and the data collection strategies were apparent and appropriate. The authors also provided data to support interpretation and elements of study relevance and transferability. However, the description of the analytical approach in these studies was unclear. Details of how the subjective meanings of participants were portrayed or handled and in what ways the deviant data were sought were missing. A critical review of the qualitative studies has been summarised in Table 4.

#### Overview of research findings

The outcomes and results of the type of interventions are summarised in Table 1 for randomised control trials and cohort and cross-sectional studies. The two following main themes emerged when data were synthesised:

- Outcome of care was generally reported as any progress in patients’ psychosocial and physical functioning (or an overall improvement in patients’ quality of life), detection of recurrence, management of comorbidities and preventive health.
- Perspectives of care were reported as patients’ satisfaction with the care provided by the general practitioner or health professionals’ views of the general practitioners’ role in providing post-treatment cancer.

### Outcomes of care

#### Physical and psychological outcomes and quality of life

Seven studies reported general practitioners’ supportive role in providing post-treatment cancer care in the context of ongoing specialists’ care [26–29, 32, 35, 43]. There were mixed results reported on quality of life benefits to patients.

Holtedahl et al. [27], in a randomised control trial investigating whether patients benefit from increased contact with their general practitioner after cancer treatment, showed that for the 81 patients who answered two sets of quality of life questionnaires, there was an improvement at 6 months in the physical, role and social function status (*p* = 0.032, 0.004 and 0.032, respectively), but this effect on the quality of life was only observed when frequent contacts occurred. For the 58 patients who answered the overall care questionnaire at randomisation and at 6 months, there was a lack of a significant improvement in their perception about care provided by their general practitioner (*p* = 0.060). However, this lack of a significant difference may have occurred due to the study inclusion criteria. The number of excluded patients was high in this study and most patients had stable disease (73 out of 78), and thus may have had little need for increased general practitioner support at the time of inclusion.

Bergholdt et al. [26] examined the involvement of general practitioners in cancer rehabilitation, with the primary outcome being the global health status of patients after 6 months. They allocated 281 patients to the intervention group and 297 to the control group (hospital care only) and found that the intervention had no statistically significant impact on the primary outcome. Adjustment for age and sex showed results similar to the unadjusted analysis. Overall, this intervention had a limited effect on the quality of life and psychological distress of patients but had a positive impact on patients’ evaluation of co-operation between primary and secondary health care sectors. A quality analysis of this study based on CONSORT revealed an adequate sample size. Although this study was powered (80%; ἀ = 0.05, *n* = 144) to detect differences between the groups on the primary outcome, process evaluation measures such as general practitioner proactivity and patient participation were not done and this may have affected the quality of life results. To improve the quality of life outcome, Bergholdt et al. [26] recommended the development of screening tools that support identification of patients with special needs and initiatives that support the general practitioners in undertaking a proactive role for patients with cancer needs.

Nielsen et al. [28] reported results similar to Bergholdt et al. [26]. In this study, a discharge summary letter detailing patients’ potential or current physical, psychological and social problems was sent to the general practitioner at the end of the treatment period by the oncologists. Patients in the intervention group were encouraged to visit their general practitioner. Patients’ attitudes to the health care services, reports on contacts with the general practitioner, health-related quality of life and performance status were evaluated at baseline and 3 and 6 months thereafter. The results of patients’ assessments of their health-related quality of life using the EORTC QLQ-C30 measure showed no statistically significant differences between the intervention and control groups, but there were improvements in quality of care offered in the intervention group.

#### Preventive health and management of other chronic illnesses

Earle and Neville in a retrospective cohort study analysed chronic comorbidities and preventive health care of cancer patients being managed by both primary care physicians and specialists. In this study, 50% of survivors (7465 patients) continued to see an oncologist in follow-up and 8% of these survivors (587 patients) saw only an oncologist. In all categories of care, patients who were followed by both oncologists and primary care physicians received the highest proportion of recommended care for the management of cancer, chronic illnesses and preventive health, followed by patients who were followed by primary care physicians. Patients who were followed only by oncologists received significantly worse preventive care compared with patients who also had a primary care physician. Survivors who did not receive care from an oncologist were less likely to undergo cancer-related procedures of surveillance colonoscopy (27.6% vs. 46.7%) and mammography (26.5% vs. 31.3%) compared with patients who saw an oncologist. Conversely, the subset of patients who were seen only by primary care physicians was more likely to receive influenza vaccination (55.2% vs. 43.6%), cervical screening (14.7% vs. 8.2%) and bone densitometry (3.9% vs. 1.1%) compared with patients who were followed only by an oncologist [13].

In an analysis by Haggstrom et al. [34] on the type of doctor speciality that cancer patients frequently visited during cancer follow-up care other than the oncologist, 16% of those on follow-up (*n* = 303) reported visiting a general practitioner. Of these, 70% had two or three other medical conditions they were being followed up for in primary care [34]. In this study, survivors were asked if they received follow-up medical tests to check for signs of other health conditions, if their doctor discussed preventive health issues such as lifestyle changes, type of diet or physical exercise. The results of this study survivors of colorectal cancer who most often saw oncologists were still significantly more likely than those who saw primary care providers to report seeing a doctor for follow-up tests and less likely to receive a physical exam. In terms of health promotion activities, colorectal cancer survivors who most often saw primary care physicians for follow-up cancer care were significantly more likely than survivors who saw specialists to report that their follow-up doctor helped with lifestyle (83% vs. 63%; *p* = 0.015) and discussed diet (70% vs. 48%; *p* = 0.005). In models adjusting for patient characteristics, oncologists were significantly less likely than primary care providers to discuss disease prevention, provide help with lifestyle and discuss diet [34].

Anvik et al. [44] explored the role of a general practitioner in post-treatment cancer care of patients recently treated and reported that patients trust their general practitioner to provide competent care, especially when they have more complex health care needs besides cancer.

## Perspectives of Care

### Patients’ perspectives

Satisfaction with care was reported both qualitatively and quantitatively in six studies [27, 28, 31, 41, 43, 44]. Holtedahl et al. [27] reported that there was a non-significant tendency towards higher satisfaction among patients whose general practitioners were involved in their care. The improvement in scores on perceptions of patients regarding their overall cancer care was evident between randomisation and 6 months (score from 55.2 to 58.9; *p* = 0.060). Furthermore, when the authors conducted a subgroup analysis comparing those patients treated with curative intent and those offered palliative treatment, this tendency was confirmed for patients treated with curative intent (62.15 vs. 46.38; *p* = 0.035) [27].

In an analysis done by Aubin et al. [32] on patients’ perceived gap between actual and expected family physician involvement in cancer care at all phases of cancer, patients preferred their family doctor to be involved in all aspects of care.

Similarly, Nielsen et al. [28] reported a statistically significant difference between the intervention and control groups at 3 months when patients’ attitudes towards co-operation (between general practitioner and oncologist) and their feeling of ‘not being left in limbo’ were assessed (*p* = 0.025). A subgroup analysis of these variables showed that men in the general practitioner-integrated programme felt less ‘left in limbo’ (*p* = 0.031) as did the younger age group (18–49 years) at both 3 and 6 months (*p* = 0.024 and *p* = 0.031), respectively. In this study, male gender (*p* = 0.007) or younger age (*p* = 0.029) were predictors of increased contact visits with a general practitioner and in the ability of the general practitioner to manage post-treatment cancer care.

Qualitative studies also reported comparable results. Hall et al. [29] modelled a shared care model and explored the views of potential patients and opinions/experiences of patients/doctors in the model, while Hudson et al. [41] explored survivor preferences of the shared care model.

Hall et al. [29] and Hudson et al. [41] revealed that patients were more receptive of general practitioner involvement in post-treatment cancer care if they were confident that the general practitioner had received extra training and support from the hospital. The shared care model was also seen as favourable to participants because of reduced waiting time and parking fees. In particular, this model was reported to be valuable to those living in the regional areas because of reduced number of hospital visits and travel logistics [29].

Five studies reported continued patient contact with the general practitioner while patients were on follow-up in the hospital [28, 31, 32, 34, 37]. Aubin et al. [32, 38], Bowman et al. [31] and Lundstrøm et al. [37] assessed family physician involvement in cancer care and found that large proportions (88%, 62% and 35%, respectively) of patients continued to visit their general practitioner informally throughout their cancer journey despite being followed up in the hospital by the specialist.

Similarly, Nielsen et al. [28] noted that patients randomised to the shared care group had an increased number of visits to their general practitioner at 3 and 6 months (*p* = 0.049 and *p* = 0.042, respectively).

### Health professionals’ perspectives

Three studies [29, 35, 44] included in this review evaluated the health professionals’ views/experiences of general practitioner involvement in post-treatment cancer care. Forsythe et al. [35], in a survey of oncologists and general practitioners, examined perceptions of shared responsibility for psychological follow-up of cancer patients. In this study, general practitioners were more likely to report shared provision for the management of physical symptoms and sole provision for health promotion and psychosocial care compared to oncologists. On the other hand, oncologists reported a shared approach regarding provision of patient psychosocial care (*p* < 0.001). Among the aspects of psychosocial care provided by general practitioners were treatment of sexual dysfunction, depression and anxiety [35]. Similarly, Wind et al. [45] explored experiences of surgeons in addressing cancer-related psychosocial problems and other non-cancer-related physical problems and reported that over 40% of the surgeons felt that these issues were beyond their field of experience.

Wind et al. and Anvik et al. reported that both general practitioners and surgeons were confident that the general practitioners can handle post-treatment cancer care in patients with a low risk of recurrence (*p* = 0.004) [45] and that the general practitioner has a place in the follow-up of many patients with cancer, including the initial phase after treatment [44].

Hall et al. [29], in a qualitative study exploring general practitioners’ and patients’ experiences/opinions of general practitioner involvement in post-treatment care, reported that general practitioners felt that their own clinical skills were improved when they received support and training. In this study, clinical skills of general practitioners were enhanced by attending training seminars and shadowing the specialist at the cancer clinics.

## Discussion and Conclusion

This review of the literature reported the outcomes of general practitioner involvement in post-treatment care alongside hospital care for cancer. Nearly all reviewed studies indicated that involving the general practitioner in the care of patients alongside specialist visits is possible and acceptable to patients. Evidence from the reviewed papers suggested that it is feasible to involve general practitioners in the care of cancer patients, provided they are equipped with the necessary skills.

This review indicated that both specialists and general practitioners were confident that the general practitioner was able to take a role in post-treatment cancer care. Most general practitioners are prepared to take a more prominent role in post-treatment cancer contingent on good specialist support. The reviewed studies showed no differences between the general practitioner and the specialist in management of patients’ physical health and general practitioners were better at recognising psychosocial issues. Patient contact with the general practitioner for supportive care was significantly associated with identification and management of psychosocial issues. Overall, general practitioners reported greater involvement in the management of psychosocial issues and shared management with the specialist on physical symptoms.

There were conflicting results from studies that examined quality of life. Overall, general practitioner involvement in follow-up care was associated with improvement in physical and social well-being of patients following cancer treatment. In studies where general practitioner involvement did not result in statistically significant improvement in quality of life, patients reported enhanced quality and coordination of care. In fact, patients who had their general practitioner involved and had other concomitant health conditions reported greater continuity of care and a less fragmented approach to managing their health.

Caution should however be exercised when interpreting such results as the study periods were relatively short in some of the reports, the measures used to assess quality of life were different and the reporting quality of each study was variable. For all studies, there were many aspects of methodological quality identified. Overall, regarding the quality of life aspect, the quality of data was generally poor and no conclusive evidence can be drawn from the collated data or narratives.

Given that not all studies included in this review performed a subgroup analysis on the effect of general practitioner involvement on patient outcomes with different types of cancer, it is plausible that the effects may differ if this model was to be applied to specific cancers. Most studies reported an overall effect of general practitioner involvement on patient outcomes (physical, psychological and social) for all cancers combined.

The reviewed studies did not provide strong evidence of the patient's role in driving the delivery of care. Most studies either mentioned patient proactive approaches as a recommendation in their summary of findings or in the methodology. In some studies, patients were encouraged to visit their general practitioner if they perceived a need to do so, and in others the general practitioner was furnished with an assessment of the patient's condition and was encouraged to invite the patient for a consultation. The quality of measures used to aid general practitioner consultation was not standardised. In some studies, general practitioners used broad questions to assess the general well-being of the patient, whereas in other studies the nurse coordinator assessed the needs of the patients, sent the report to the general practitioner and encouraged the general practitioners to contact patients. The deployment of a validated questionnaire detailing possible needs of the patients and how this assessment was used to encourage a consultation with the general practitioner was limited. In fact, Bergholdt et al. [26] recommended the use of a screening tool or a decision support tool to identify and address patient needs.

## Study Strengths and Limitations

The small number of studies identified for this review may be due to a number of reasons. Ongoing involvement of the general practitioner in post-treatment cancer care was not clearly described in the literature. In some cases, this was described as ‘formal’ shared care where different roles of the general practitioner and those of the specialist were clearly delineated, whereas in other studies this was described as ‘informal’ shared care where patients continued to visit their general practitioner informally while still on scheduled visits with the specialist. However, database searches were supplemented by a hand search from the list of references of the identified papers and systematic reviews.

Finally, heterogeneity of the study methods, outcome measures and analytical approach of various studies meant that no data could be pooled in a meta-analysis.

## Recommendations

To improve patient outcomes in this approach, the design and testing of validated measures that support identification of patients who may benefit from general practitioner involvement while still on ongoing care from the specialist may be helpful. Additionally, devising and deploying initiatives that encourage and facilitate patients to consult their general practitioner first about what they believe are the needs or symptoms of recurrence should be considered. Applying this model of post-treatment cancer care to patients with a specified cancer would clarify whether the outcomes would be different.

This review of the literature demonstrates that general practitioner involvement alongside specialist care for cancer patients has not been robustly explored despite some of the studies concluding that they are feasible and acceptable to patients.

## Transparency

### Declaration of funding

This project was funded through a Curtin University internal grant.

### Declaration of financial/other relationships

I.N., M.J., A.M., and J.H. have disclosed that they have no significant relationships with or financial interests in any commercial companies related to this study or article.

## Figures and Tables

**Table 1.**
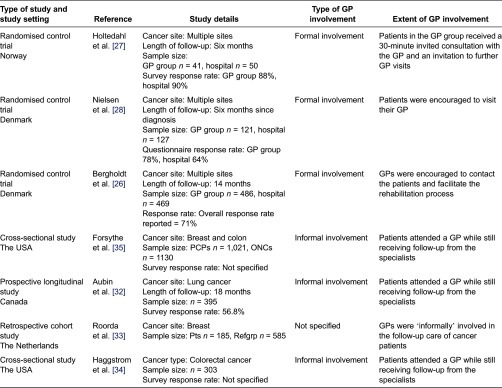
Results indicating type of GP involvement in cancer care

**Table 2.**
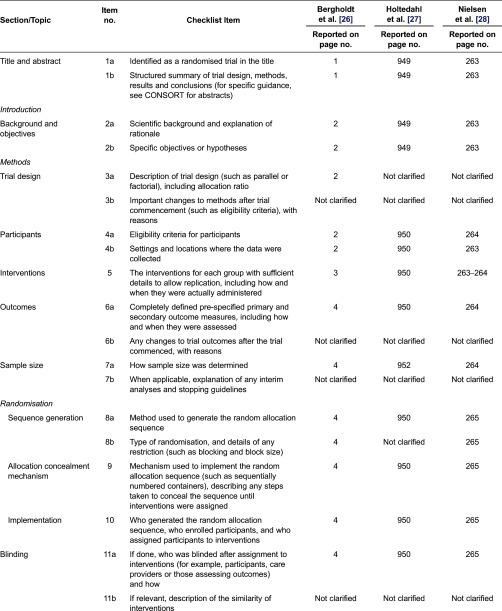

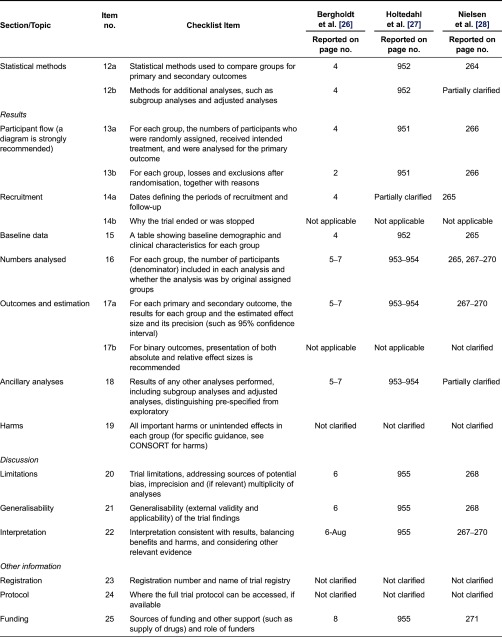
Critical review of randomised control trials assessing GP involvement in cancer care using CONSORT

**Table 3.**
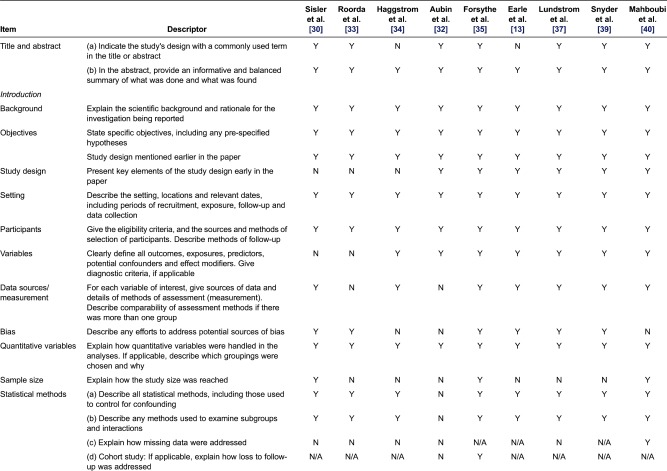

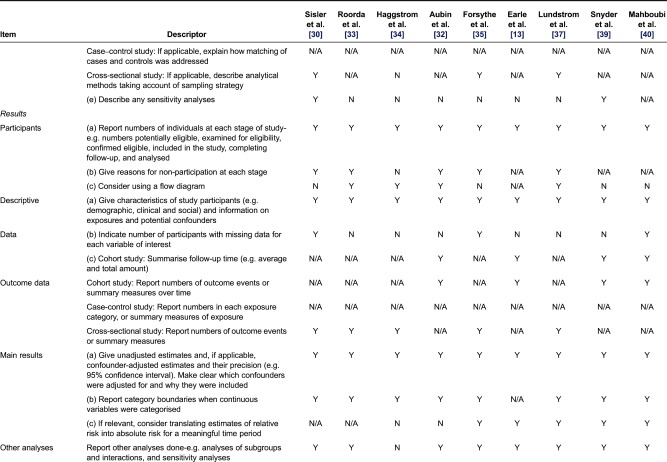

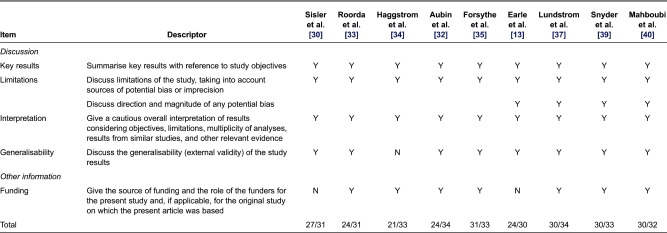
Critical review of observational studies assessing GP involvement in cancer care using STROBE

**Table 4.**
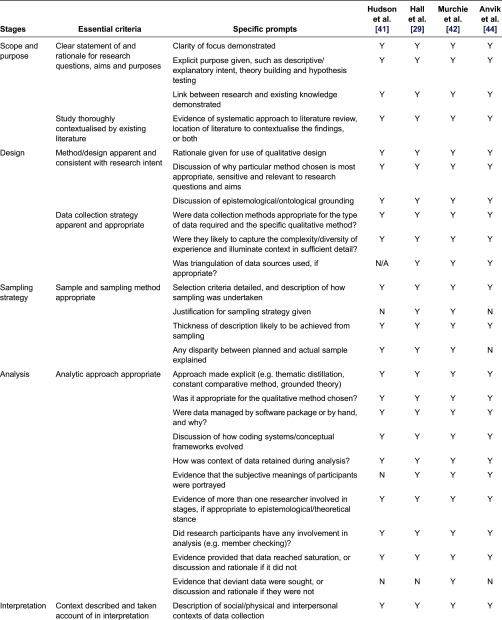

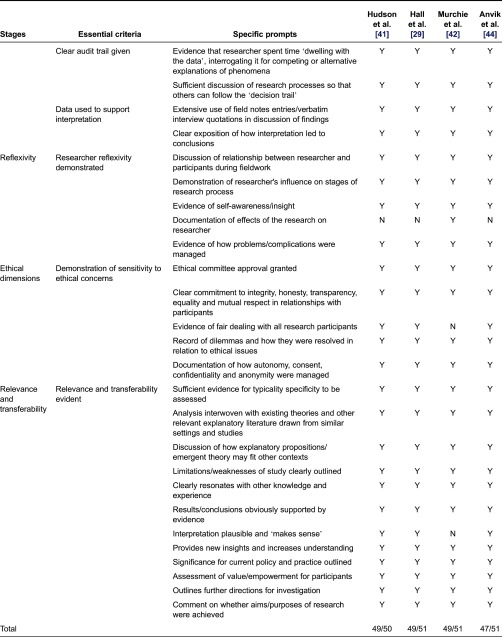
Critical review of qualitative studies assessing GP involvement in cancer care using Walsh and Downie criteria
